# Anti-Inflammatory Activity of Vacuum Distillate from *Panax ginseng* Root on LPS-Induced RAW264.7 Cells

**DOI:** 10.4014/jmb.2312.12001

**Published:** 2024-01-14

**Authors:** Chanwoo Lee, Seul Lee, Young Pyo Jang, Junseong Park

**Affiliations:** 1 College of Pharmacy, Kyung Hee University, Seoul 02447, Republic of Korea; 2 Department of Engineering Chemistry, Chungbuk National University, Cheongju 28644, Republic of Korea

**Keywords:** Anti-inflammation, ginseng distillate, MAPK pathway, NF-κB pathway

## Abstract

*Panax ginseng* has been widely applied as an important herb in traditional medicine to treat numerous human disorders. However, the inflammatory regulation effect of *P. ginseng* distillate (GSD) has not yet been fully assessed. To determine whether GSD can ameliorate inflammatory processes, a GSD was prepared using the vacuum distillation process for the first time, and the regulation effect on lipopolysaccharide-induced macrophages was assessed. The results showed that GSD effectively inhibited nitric oxide (NO) formation and activation of inducible nitric oxide synthase (iNOS) mRNA in murine macrophage cell, but not cyclooxygenase-2 production. The mRNA expression pattern of tumor necrosis factor alpha and IL-6 were also reduced by GSD. Furthermore, we confirmed that GSD exerted its anti-inflammatory effects by downregulating c-Jun NH_2_-terminal kinase (JNK) phosphorylation, the extracellular signal-regulated kinase phosphorylation, and signaling pathway of nuclear factor kappa B (NF-κB). Our findings revealed that the inflammatory regulation activity of GSD could be induced by iNOS and NO formation inhibition mediated by regulation of nuclear factor kappa B and p38/JNK MAPK pathways.

## Introduction

Inflammation is an indispensable response of inborn immune processes and various biological systems that protect the body from infections caused by external pathogenic agents [1, 2]. Many systemic diseases, such as arteriocapillary sclerosis, bowel disease, pneumonia, hyper-lipidemia, and multiple sclerosis, emerge accompanied by severe inflammatory responses [3, 4]. Macrophages are crucial immune cells in charge of inborn cellular immunity involved in the defense mechanism against external irritants. They are induced by lipopolysaccharides (LPSs), a major part of the gram-negative bacterial membrane, and adjust inflammation by discharging pro-inflammatory transmitter substances, such as inducible nitric oxide synthase (iNOS), prostaglandin E2 (PGE2), cyclooxygenase 2 (COX-2), tumor necrosis factor (TNF)-α, inter-leukin (IL)-1β, and IL-6 [4-6]. Imbalanced production of these transmitter substances results in chronic inflammatory disorders, such as various types of cancer, arteriosclerosis, diabetic nephropathy, and neurodegenerative diseases [7]. The pathological effects of inflammation on humans emphasize the need to develop safe and effective medicines that target various bio-chemical processes to reduce the inflammatory response.

*Panax ginseng*, which is a perennial plant affiliated with the *Panax* genus of the family Araliaceae, has been widely applied for a long time as an important herb in traditional medicine to treat numerous human disorders in East Asia [8]. The pharmacological activity of ginseng arises from the complex action of its various ingredients, including ginsenosides, acid glucan, glycopeptides, phytosterols, and polyacetylenes [9-11]. Among the major ginseng products, white ginseng is usually produced by means of an air-drying process involving freshly harvested ginseng, whereas red ginseng is produced by steaming and drying fresh ginseng. Ginseng is consumed either in a fresh form (3,761 tons, 18.0%) or in processed form, such as red (16,376 tons, 78.4%) and white ginseng (736 tons, 3.5%) in Korea [12]. Most studies on ginseng have focused on the efficacy of extracts from the dried root. However, 60-70% of the mass of harvested ginseng roots comprises unbound water containing volatile components [13]. This unbound water component, which can be called ginseng distillate (GSD), is lost into the air during the process of drying ginseng for most commercial purposes [14, 15]. Based on the known functions of ginseng, it is crucial that GSD is effectively secured, as thousands of tons disappear every year.

Vacuum distillation technology is typically used to separate active compounds such as flavonoids, vitamins, and lipid compounds [16-18]. The available active compounds in natural products can be obtained with higher purity and content when separated under reduced pressure conditions than under normal conditions [19]. This may be because the decomposition and oxidation of compounds that are sensitive to heat and are easily oxidized can be suppressed when maintaining a relatively low temperature environment through reduced pressure. Additionally, the vacuum environment provides an ideal state for dynamic processes to promote dissolution and diffusion of active components [20]. However, vacuum distillation has rarely been applied to separate the unbound water from ginseng, and the biological activities of GSD have not yet been investigated. In this study, we aimed to use vacuum distillation to isolate GSD and investigate its properties. The assessment of the anti-inflammatory level of the GSD on NO formation, inflammatory gene expression adjusting, and inflammatory signaling cascades revealed that GSD exerts anti-inflammatory activity by iNOS and NO production suppression linked with NF-κB and p38/JNK MAPK pathway modulation.

## Materials and Methods

### GSD Preparation

A vacuum distillation unit was used to produce distillate of fresh ginseng root. The apparatus comprised a vacuum oven and a condenser pipe combined with a vacuum pump at the bottom. The pressure maintenance was adjusted using the controllable vacuum pump. A pump with a circulating cooler fluid was operated at 0°C with 50% aqueous ethanol solution to prevent the loss of volatility during the operation. Fresh ginseng root (500 g; collected from Eumseong, Republic of Korea) was placed in a vacuum oven set to 60°C and distilled under vacuum conditions for 12 h. GSD was obtained with a yield of approximately 70.0 wt%. The GSD was then stored in a brown bottle and refrigerated for subsequent experiments.

### Cell Culture and MTT Cell Viability Assay

The murine macrophage RAW264.7 cell line was purchased from Korean Cell Line Bank (KCLB 40071, Seoul, Korea). The cells were cultured in plastic dishes containing Dulbecco’s modified Eagle’s medium (DMEM; Hyclone, USA) supplemented with 10% fetal bovine serum (Hyclone), 100 U/ml penicillin, and 100 μg/ml streptomycin in a 5% CO_2_ incubator. All cells grown at a density of approximately 80–90% in the experimental process were sub-cultured. Cell viability was assessed using the MTT assay. Briefly, 10^5^ RAW264.7 cells/well were incubated with various concentrations of GSD (0–30%) for 24 h, and then the medium was discarded. The cells were washed and treated with 50 μl of MTT, after which the plates were incubated in the dark for 3 h. The resultant formazan crystals were dissolved in 100 μl of DMSO, and the absorbance was measured at 550 nm. The control group of untreated cells was considered as having 100% viable cells. Results are expressed as the percentages of viable cells, compared with those in the control group.

### NO Production Assay

Murine macrophages were pre-incubated in 96-well plates (10^5^ cells/well). After initial incubation, LPS (1 μg/ml) and GSD (0–30%) were added, and the culture was incubated for 24 h. Next, 100 μl of supernatant was mixed with 100 μl of Griess reagent, and the culture was again incubated for 10 min. The absorbance was measured at 540 nm using a microplate reader (BioRad, USA), and the quantity of nitrite was calculated based on the standard curves of sodium nitrite.

### Reverse Transcription-PCR

TRIZOL reagent (Molecular Research Center, USA) was used to isolate total RNA. Single-stranded cDNA was synthesized from 2 μg of total RNA using RT premix (Bioneer, Korea). PCR was carried out using PCR premix (Bioneer) and several primers. The primers for iNOS were TAGTTTCCAGAAGCAGAATGTGACC and AGAGCAATGACTCCAAAGTAGACCTG; for COX-2 were GGAGAGACTATCAAGATAGT and ATGGTC AGTAGACTTTTACA; for TNF-α were TACCTTGTCTACTCCCAGGTTCTCTTC and AGAGCAATGACT CCAAAGTAGACCTG; for IL-6 were AGCCAGAGTCCTTCAGAGAGA and TAACGCACTAGGTTTGCCGAG; and for β-actin were TGGAATCCTGTGGCATCCATGAAAC and TAAAACGCAGCTCAGTAACAGTCCG. PCR products were separated on 1% agarose gels and stained with 5 μg/ml of ethidium bromide. Band intensities were quantified using ImageJ 1.47 software (NIH, USA).

### Western Blot Assay

Murine macrophages treated with GSD (0–30%) and LPS (1 μg/ml) for 24 h were lysed with lysis buffer. The cell lysates were centrifuged at 13,400 ×*g* to remove the cell membrane material. The protein concentration was measured using the Bradford reagent (BioRad). Protein samples were separated on 10% SDS–polyacrylamide gel electrophoresis and then transferred onto PVDF membranes (BioRad). After blocking nonspecific sites with 5%skim milk (Fluka, Switzerland) in 0.1% Tris-buffered saline (TBS)-Tween 20, the membranes were incubated with anti-mouse NF-κB p50, NF-κB p-p65, NF-κB p65, ERK1/2, p-ERK1/2, JNK, p-JNK, and β-actin antibodies in TBS (1:500) for 2 h. The membranes were further incubated for 1 h with horseradish peroxidase-conjugated anti-mouse IgG and antirabbit IgG (1:2000). The immune-active proteins were detected using an enhanced chemiluminescence detector. The signal intensity of each protein band was quantified using ImageJ 1.47 software.

### GC-MS

GC-MS analysis was performed using an Agilent 7890B gas chromatograph (Agilent Technologies, USA) interfaced to an Agilent 5977B mass selective detector (70 eV, electron impact ionization mode) and equipped with an Ultra-2 cross-linked capillary column (Agilent Technologies). The carrier gas was helium at a flow rate of 0.5 ml/min in linear velocity mode. The sample (1 μl) was introduced using the Agilent 7693A auto-injector and Agilent G4513A auto-sampler in the split-injection mode (10:1). The range of the scanned mass was 50–700 u at a rate of 0.99 scan/s.

### Statistical Analysis

All data are expressed as means ± SD of at least three separate experiments, each of which were carried out in triplicate. Statistical analyses were performed using Microsoft Excel software (unpaired Student’s *t*-test, *p < 0.01).

## Results

### Cell Viability Assay of GSD and Its Effect on NO Formation Stimulated by LPS

**Fig. 1:**
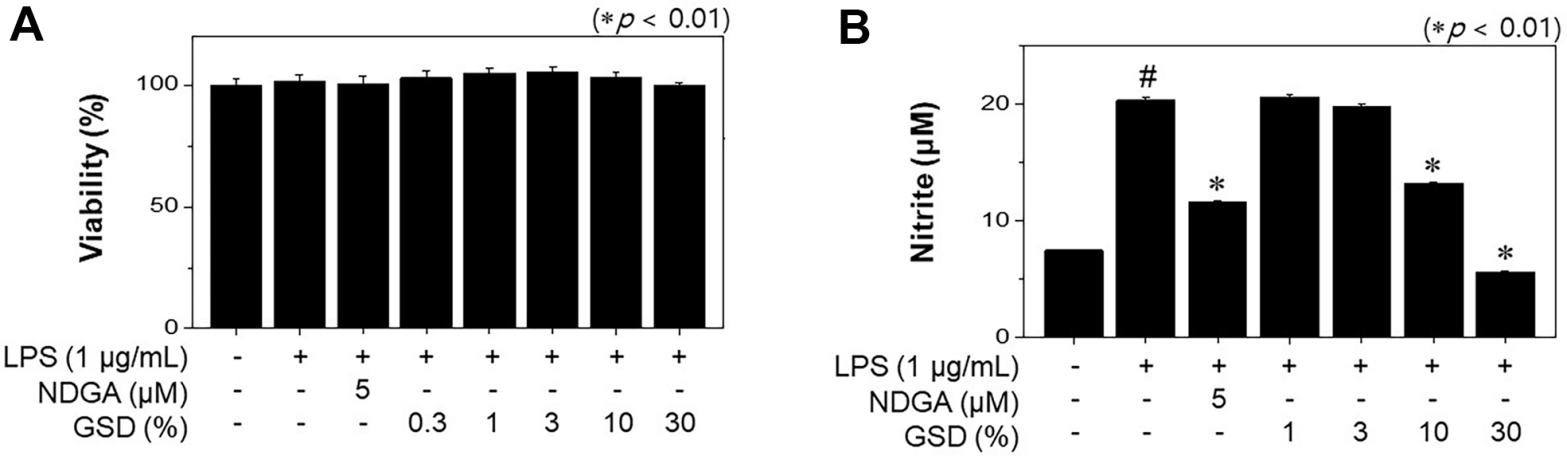
Effects on cell viability and nitric oxide (NO) production by ginseng distillate (GSD) in lipopolysaccharide (LPS)-stimulated RAW264.7 cells. (**A**) Cell viability was assessed in cells stimulated with LPS (1 μg/ml) in the presence of GSD for 24 h. (**B**) NO production was determined using the Griess reagent method. Data represent the mean ± SD of triplicate experiments. Significance was determined versus samples untreated (#*p* < 0.01) or sample only treated cells (*p < 0.01).

The potential cytotoxicity of GSD was assessed using MTT assay after cells were incubated with or without LPS treatment. Fig. 1A shows that macrophage viability was not affected until treatment concentrations of GSD were 0.3, 1, 3, 10, or 30%. Thus, no cytotoxic effects of GSD were observed on the RAW264.7 cells. Subsequent experiments were conducted using GSD at concentrations between 3 and 30%. To investigate whether GSD could regulate NO formation, we assessed NO release in LPS-stimulated RAW264.7 macrophages after GSD treatment. The LPS-treated cells stimulated significant NO release compared to the control cell group (Fig. 1B). However, GSD-treated cells controlled NO formation in a concentration-dependent manner; in particular, when dosed with GSD at a concentration of 30%, the NO formation was suppressed to almost the initial level (6.45 ± 0.02 μM). Our results show that concentrations of 10 and 30% GSD suppress NO formation in LPS-stimulated macrophages.

### Effect of GSD on iNOS and COX-2 mRNA Expression Pattern in LPS-Stimulated RAW264.7 Macrophages

**Fig. 2:**
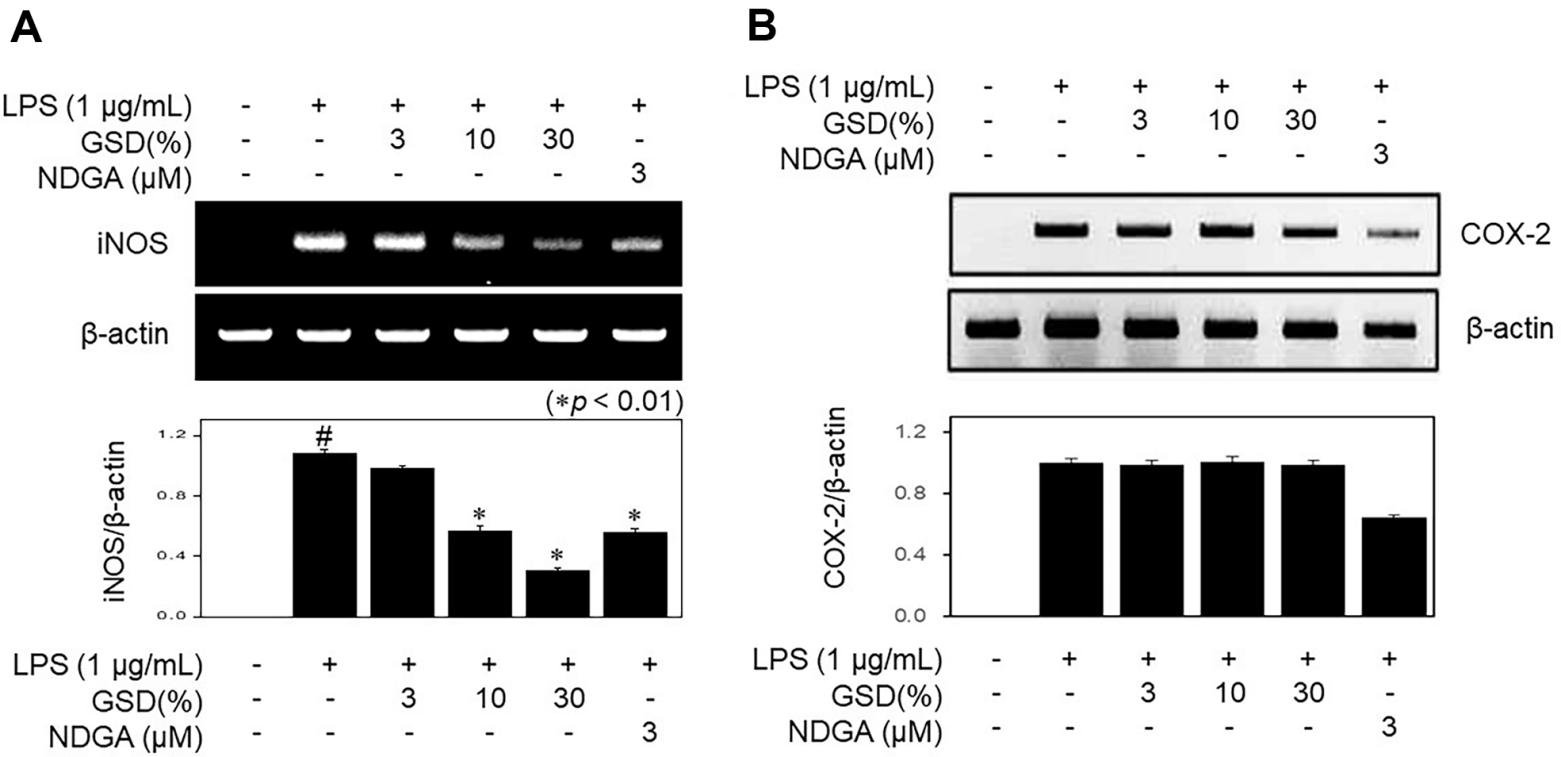
Effects on (**A**) inducible nitric oxide synthase (iNOS) and (**B**) cyclooxygenase-2 (COX-2) mRNA expression by GSD in lipopolysaccharide (LPS)-stimulated RAW264.7 cells. The cells were treated with GSD (3– 30%) in the presence of LPS (1 μg/ml) for 24 h. RAW264.7 cells were treated simultaneously with 3 μM of nordihydroguaiaretic acid (NDGA) and LPS. For reverse transcription-polymerase chain reaction (RT-PCR) analysis of the gene expressions of iNOS and COX-2, total RNA was prepared. β-Actin was used as the internal control for RT-PCR. Significance was determined versus samples untreated (#*p* < 0.01) or sample only-treated cells (*p < 0.01).

iNOS, a pro-inflammatory protein, produces NO, which has a significant impact on a variety of acute and chronic inflammation disorders. Fig. 2 shows the efficacy of GSD on the mRNA expression patterns of iNOS and COX-2, which mediate NO and PGE2 synthesis. The levels of iNOS expression were significantly suppressed under exposure to concentrations of 10 and 30% of GSD, compared to positive control cells. In contrast, GSD did not induce significant changes in COX-2 expression at these doses. The LPS-induced iNOS mRNA expression was also suppressed by the iNOS inhibitor nordihydroguaiaretic acid (NDGA).

### Effect of GSD on Pro-Inflammation Cytokine Formation

**Fig. 3:**
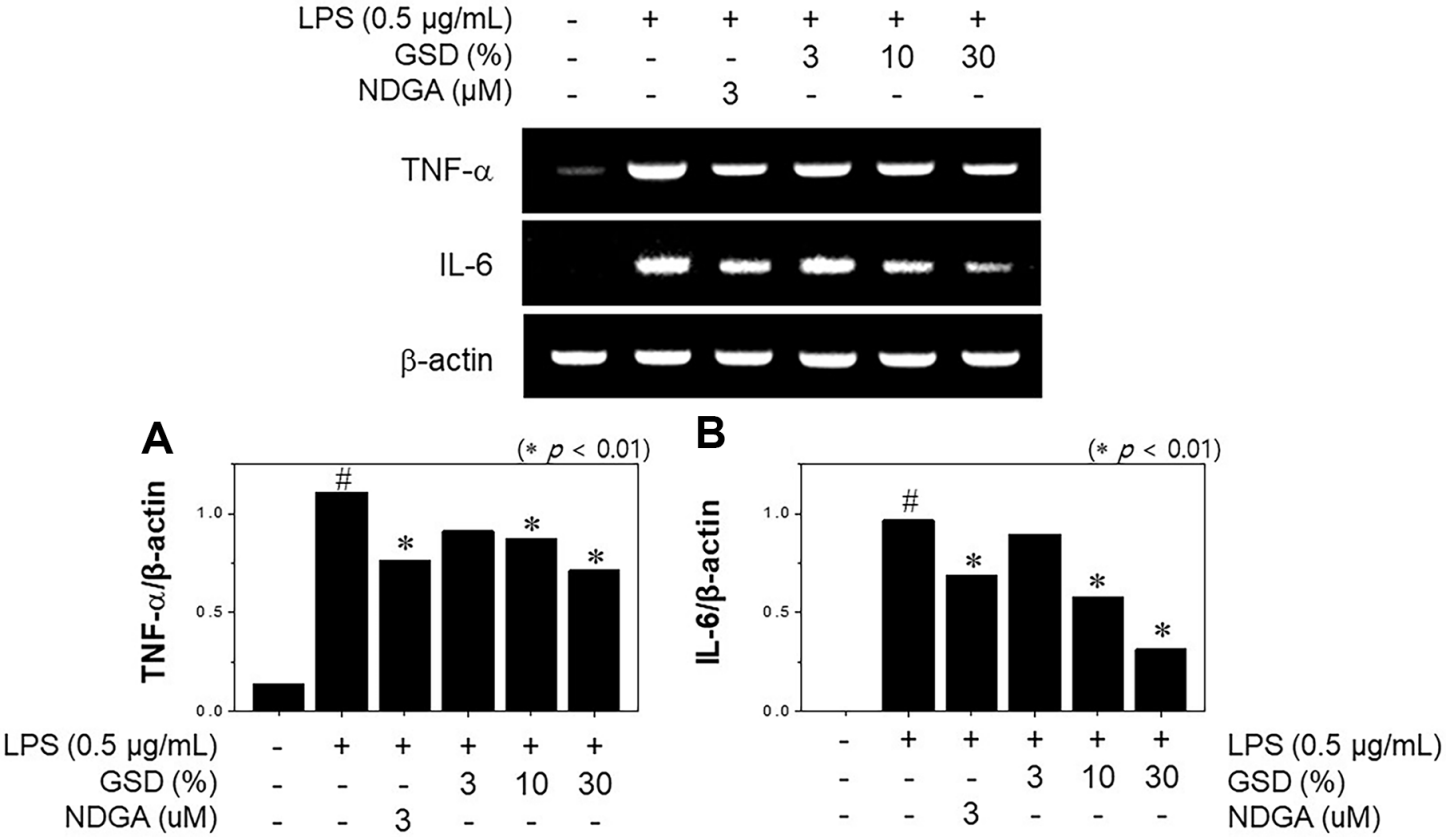
Inhibitory effect of GSD on mRNA expression. (**A**) Tumor necrosis factor-(TNF)-α and (**B**) interleukin (IL)-6 in LPS-stimulated RAW264.7 cells. The cells were treated with GSD (3–30%) in the presence of LPS (1 μg/ml) for 4 h. RAW264.7 cells were treated simultaneously with 3 μM of nordihydroguaiaretic acid (NDGA) and LPS. For RT-PCR analysis of the gene expressions of iNOS and COX-2, total RNA was prepared. β-Actin was used as internal control for RT-PCR. Significance was determined versus samples untreated (#*p* < 0.01) or sample only-treated cells (*p < 0.01).

During inflammatory responses, transmitters such as pro-inflammation cytokines can be detected in excess. Several pro-inflammation cytokines, including TNF-α and IL-6, play key roles in LPS-induced murine macrophages [21]. To evaluate the anti-inflammation potential of GSD, we assessed the pro-inflammation cytokine formation in response to LPS-stimulated inflammation. As an outcome, the mRNA expression levels of pro-inflammation cytokines were suppressed in a concentration-dependent manner (Fig. 3). TNF-α levels in LPS-induced murine macrophages were markedly reduced after concentrations of 10 and 30% GSD were applied (Fig. 3A), whereas the basal level of the cytokines appeared to be normal in macrophages. Notably, the mRNA expression of IL-6 decreased by 67.33% when a treatment concentration of 30% GSD was applied (Fig. 3B). Taken together, our results show that the anti-inflammation potential of GSD might have appeared when iNOS mRNA expression and NO release, but not COX-2 expression, are reduced.

### Effect of GSD on NF-κB Signaling in LPS-Stimulated RAW264.7 Macrophages

**Fig. 4:**
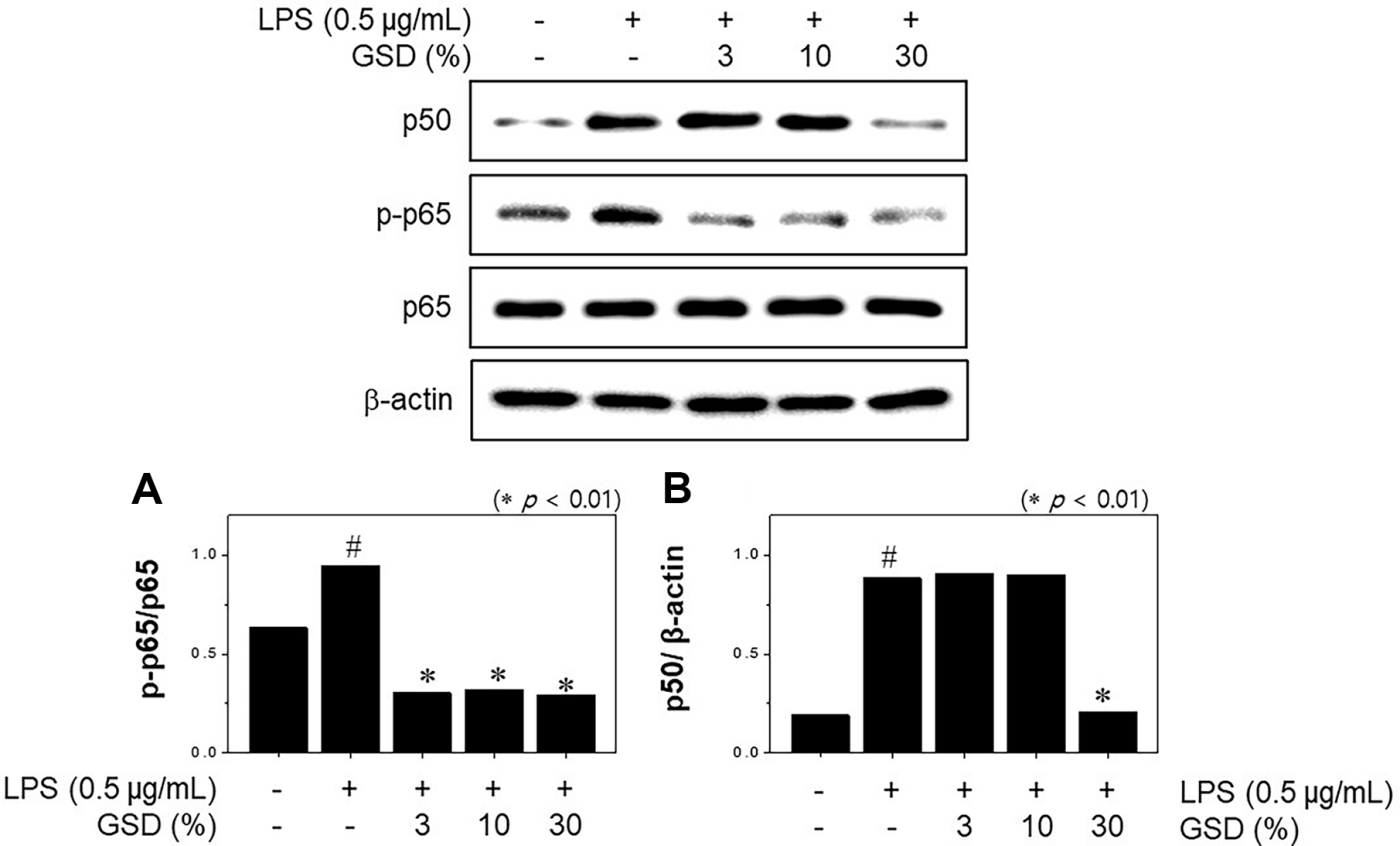
Effects of GSD on the activation of NF-κB signaling pathways in LPS-induced RAW264.7 cells. Cells were pretreated with GSD and induced with LPS (1 μg/ml) for 1 h. The protein expression levels of p50 (**A**), p-p65 (**B**), and p65 were measured by western blotting from total cell lysates. Significance was determined versus samples untreated (#*p* < 0.01) or sample only-treated cells (*p < 0.01).

Considering that NF-κB is primary for iNOS activation, we applied western blot assay to measure the expression and phosphorylation pattern of NF-κB subunits p65 and p50. p65 expression and phosphorylation were increased in macrophages upon LPS treatment; however, p65 phosphorylation was substantially suppressed by GSD administration. GSD concentration of 30% significantly inhibited p50 expression. However, administration of GSD at different concentrations did not affect LPS-stimulated p50 expression (Fig. 4).

### Effect of GSD on MAPK Signaling

**Fig. 5:**
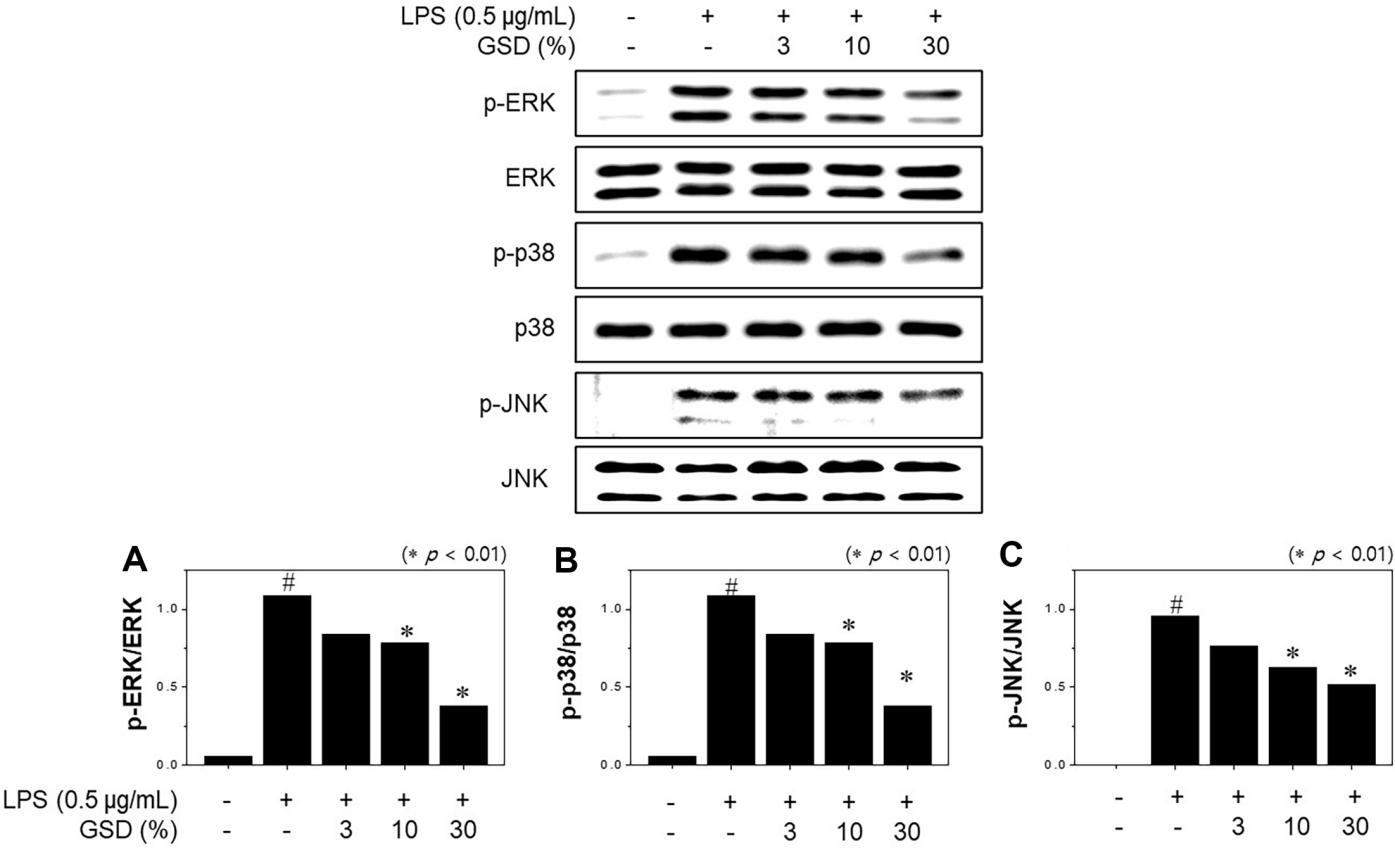
Effect of GSD on the phosphorylation of MAPK cascade (p-ERK1/2 (**A**), p-p 38 (**B**), and p-JNK (**C**)) in LPS-stimulated RAW264.7 cells. Cells were pretreated with GSD and induced with LPS (1 μg/ml) for 1 h. Significance was determined versus samples untreated (#*p* < 0.01) or sample only-treated cells (*p < 0.01).

Phosphorylation of the mitogen-activated protein kinase (MAPK) family is a primary pathway affecting LPS-induced formation of inflammatory factors. MAPKs, such as ERK1/2, JNK, and the p38 subfamily, are known to play central roles in signaling pathways and induce NF-κB activation. To examine the potential of GSD on MAPK phosphorylation, phosphorylation of JNK, ERK, and p38 was measured by western blot assay. Fig. 5 shows that the phosphorylation of JNK, ERK, and p38 was increased by LPSs, but these proteins were markedly downregulated in a concentration-dependent manner by GSD administration, compared to the LPS-only treatment group.

### Active Compounds Detected by Gas Chromatography–Mass Spectrometry Analysis

**Fig. 6:**
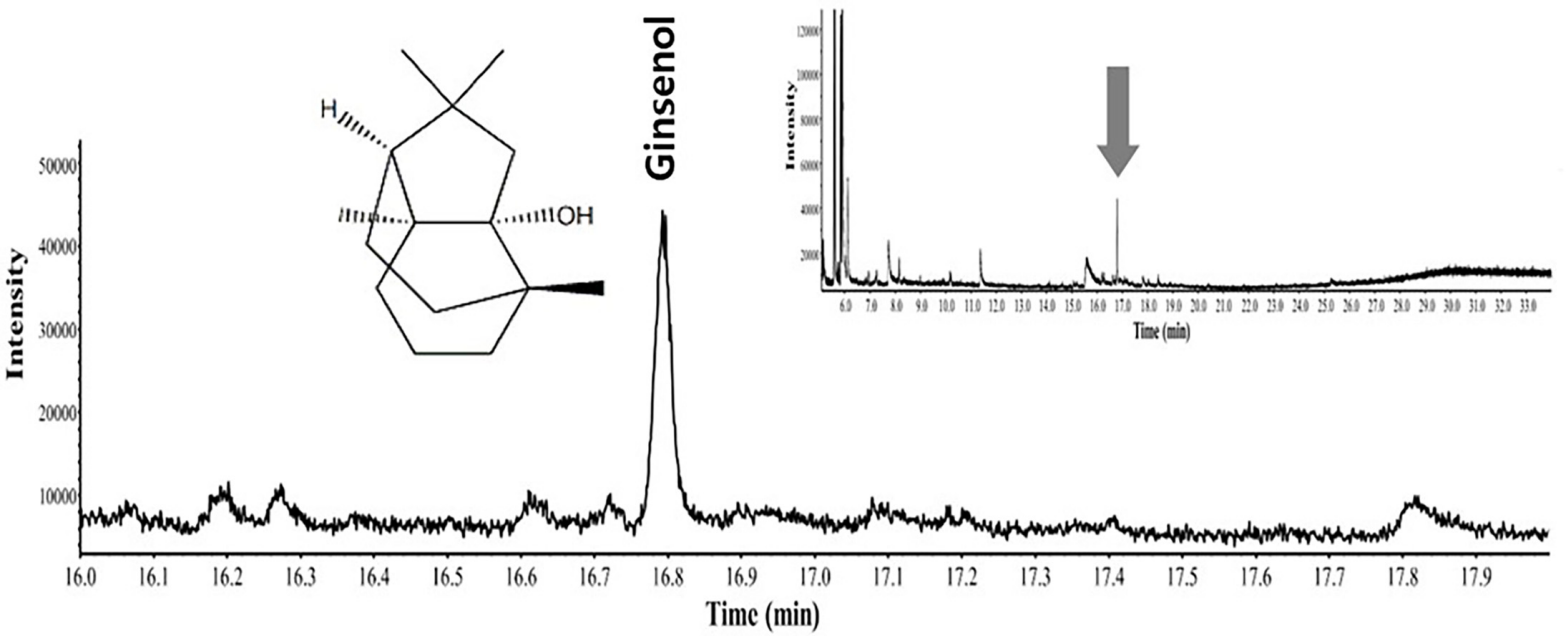
GC–MS chromatogram of GSD.

Fig. 6 shows a representative chromatogram of GSD. As a result of gas chromatography-mass spectrometry (GC-MS), we confirmed that GSD had a small number of components appearing as peaks, and the component with the highest content was ginsenol.

## Discussion

*P. ginseng* has long been applied as an herbal medicine in East Asia, and its pharmacological active compounds exhibit various activities, such as antioxidant, anti-inflammatory, and anticancer effects. Extraction and separation methods, such as solvent extraction, ultrasound, and microwaves, are used to separate the effective components of ginseng, and the various efficacies of raw materials manufactured in this way have been verified [22-24]. However, there are no confirmed cases of GSD isolated via vacuum distillation. In this study, we investigated the underlying mechanism of the anti-inflammatory effect of GSD in LPS-stimulated RAW264.7 macrophages.

Activation of macrophages by LPSs, which are an outer membrane material of gram-negative bacteria, stimulates the formation and discharge of components involved in the launch of inflammation, including cytokines, NO, and pro-inflammation enzymes. [25]. Accumulating evidence indicates that NO is involved as an inflammation initiator [26]. NO has key functions as a signaling molecule in various physiological processes, and high concentrations of NO formed by iNOS can induce inflammation [27]. Our results show that GSD administration suppressed LPS-induced iNOS mRNA expression and NO formation to levels similar to positive controls. Moreover, excessive NO production is associated with iNOS overexpression. Therefore, selective inhibitors of iNOS in inflammatory macrophages can be applied as effective treatments for inflammation disorders. In this study, GSD was confirmed to be a very effective substance in suppressing iNOS mRNA expression and NO formation. The inhibitory efficacy shown by GSD was not due to cytotoxicity. This is because treatment with GSD at the tested concentrations that showed efficacy did not result in a significant decrease in cell viability.

In addition to NO, PGE2 discharged from immune cells plays a key role in various inflammatory responses stimulated by COX-2 expression. NO formed by iNOS during the inflammatory response leads to COX-2 upregulation via crosstalk between NO, COX-2, and PGE2 [28]. Notably, GSD did not suppress the COX-2 mRNA expression in this study. Therefore, these data suggest that GSD shows selective anti-inflammation activity, resulting in iNOS expression being downregulated, but not COX-2 expression. Ginseng extracts prepared by various methods are reported to exhibit anti-inflammation activity by their suppression of both iNOS and COX-2 expressions [29]. This effect comes mostly from ginsenosides, the main ingredient in ginseng [7]. The anti-inflammatory effect of GSD was shown via iNOS and NO but not COX-2. This result might be explained by the properties of herbal extracts. It may also be attributed to the differences occurring during the GSD manufacturing process, which when carried out using the vacuum distillation method does not involve ginsenosides, and therefore appears to be a result of the compositions of other components. A similar effect was observed in extracts from *Buddleja officinalis* and *Thuja orientalis*, where iNOS activity was strongly inhibited, but COX-2 activity was weakly inhibited [30, 31].

LPS-activated macrophages secrete pro-inflammatory cytokines, such as TNF-α and IL-6, which play key roles in anti-inflammatory responses [32]. TNF-α is an important mediator that upregulates the expressions of inflammatory molecules to improve the inflammatory response. IL-6 also has pro-inflammatory properties and is considered to play an important role in the immune response [33]. In general, inhibiting TNF-α and IL-6 is considered an effective therapeutic solution against inflammation disorders. This study suggests that GSD suppresses the mRNA levels of TNF-α and IL-6 in a concentration-dependent manner in LPS-stimulated RAW264.7 macrophages. The anti-inflammatory effect of GSD may be exerted by its suppression effect on pro-inflammation cytokines, such as IL-6 and TNF-α, as well as the hallmarks of inflammation, NO, and partly by NF-κB signaling. NF-κB consists of p50 and p65 subunits and binds to the inhibitor of NF-κB (IκB), which releases free NF-κB. Once initiated, NF-κB subunit p65 separates from its inhibitory protein IκB and migrates from the cytoplasm to the nucleus, where it may lead to the transcription of specific target genes such as IL-6 and TNF-α [34]. In this investigation, the inhibition of the p50 subunit and of p65 phosphorylation, along with nuclear translocation by GSD, demonstrated that the anti-inflammatory response of GSD is related with NF-κB signaling.

MAPKs, including p38, JNK, and ERK1/2, play key roles in inducing cytokine production and positively affect NF-κB activation. The LPS stimulation of murine macrophages has been known to induce the phosphorylation and activation of p38, JNK, and ERK1/2 [35]. We examined the potential effect of GSD on LPS-induced phosphorylation of MAPKs in RAW264.7 cells. The results showed that LPS treatment stimulated the phosphorylation of p38, JNK, and ERK1/2. Furthermore, pretreatment of GSD considerably suppressed phosphorylation of p38, JNK, and ERK1/2. Collectively, these results suggest that GSD inhibited LPS-induced pro-inflammatory cytokine formation by preventing NF-κB and MAPK activation.

**Fig. 7:**
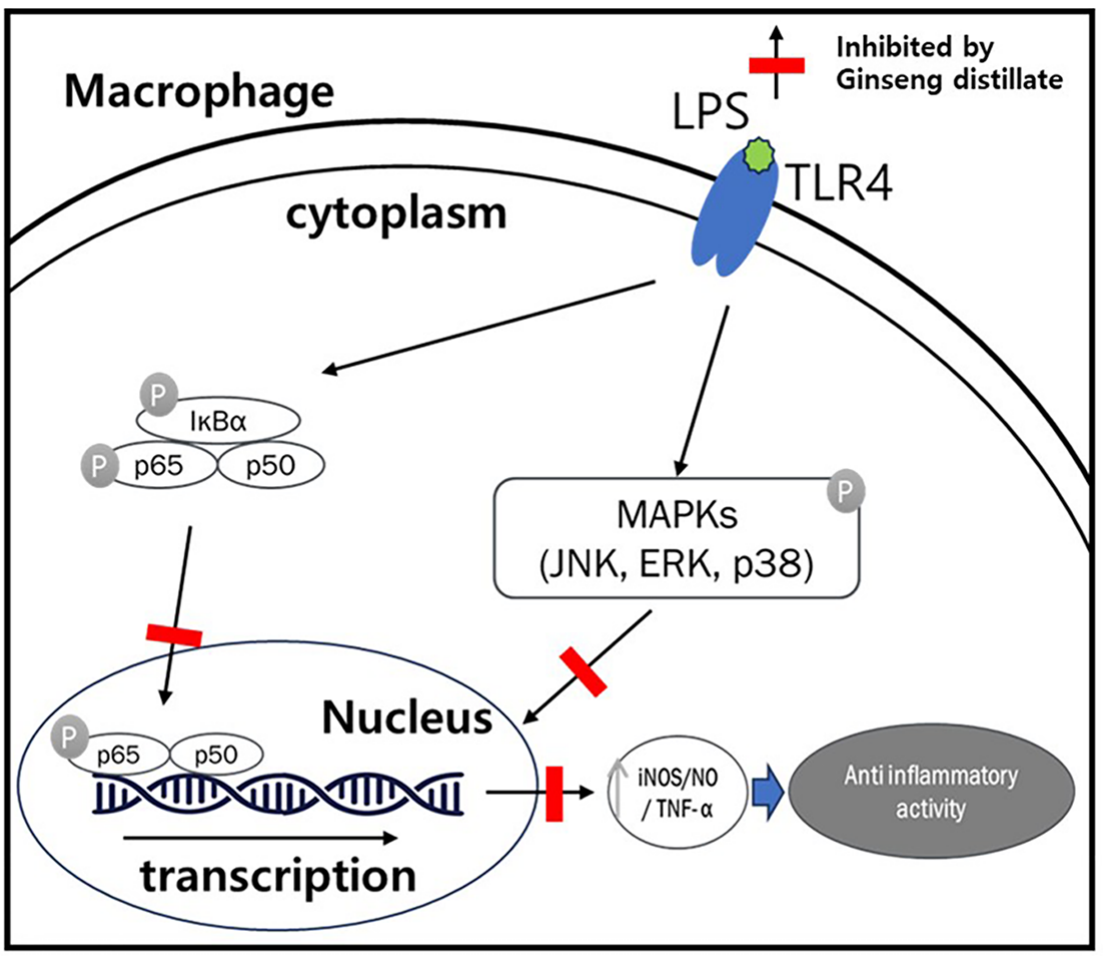
Schematic of the suppressive effects of GSD on the inflammatory response via modulating NF-κB and the MAPK signaling pathway in LPS-stimulated RAW264.7 cells.

In summary, our data demonstrated that GSD exerts an anti-inflammatory effect, which might be attributable to the inhibition of iNOS mRNA expression, by interfering with NF-κB and MAPK signaling pathways (Fig. 7). Although GSD clearly showed anti-inflammatory effect on murine macrophages in vitro in the current study, the experimental cell line was not human-derived. Therefore, we plan to perform further experiments using human-derived cells to confirm the anti-inflammatory efficacy of GSD.
